# Fatal acute pulmonary oedema and acute renal failure following multiple wasp/hornet (Vespa affinis) stings in Sri Lanka: two case reports

**DOI:** 10.1186/1752-1947-8-188

**Published:** 2014-06-13

**Authors:** Keerthi Kularatne, Thamara Kannangare, Ajith Jayasena, Aruni Jayasekera, Roshitha Waduge, Kosala Weerakoon, Senanayake AM Kularatne

**Affiliations:** 1 District General Hospital, Matale, Sri Lanka; 2 Department of Pathology, Faculty of Medicine, University of Peradeniya, Peradeniya, Sri Lanka; 3 Department of Parasitology, Faculty of Medicine and Allied Sciences, Rajarata University of Sri Lanka, Saliyapura, Sri Lanka; 4 Department of Medicine, Faculty of Medicine, University of Peradeniya, Peradeniya, Sri Lanka

**Keywords:** Acute renal failure, Pulmonary oedema, Sri Lanka, *Vespa affinis*

## Abstract

**Introduction:**

*Vespa affinis* is a hornet widely distributed in Sri Lanka and it is responsible for the highest number of deaths related to Hymenoptera stings. Apart from the early reactions, victims often die in hospital many hours later due to complications such as myocardial infarction and multiple organ failure. Increased microvascular permeability and acute pulmonary oedema as the primary pathology is less known in hornet envenoming.

**Case presentation:**

Here, we report clinical and postmortem findings of two Sinhalese patients, a 48-year-old husband and his 46-year-old wife, who both died following a massive attack by hornets 32 hours and 9 hours after the incidence respectively. At postmortem examination, both patients had pleural effusions, acute pulmonary oedema and red cell casts in their urine. Their coronary arteries and histology of myocardium were normal.

**Conclusion:**

Early recognition of acute pulmonary oedema in hornet stings is needed with implementation of crucial treatments to avert deaths.

## Introduction

**Figure 1:**
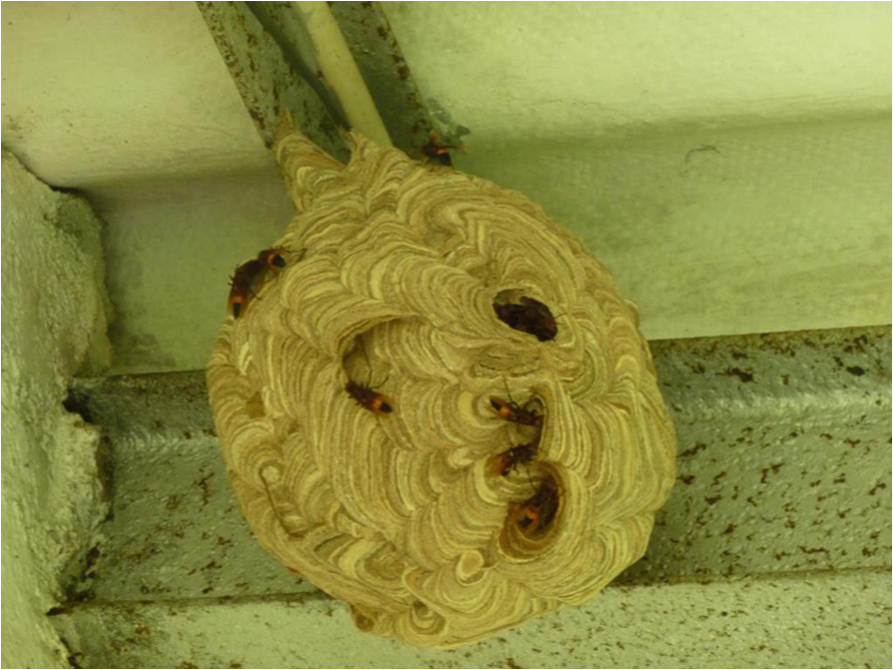
A hornet nest in a public place.

**Figure 2:**
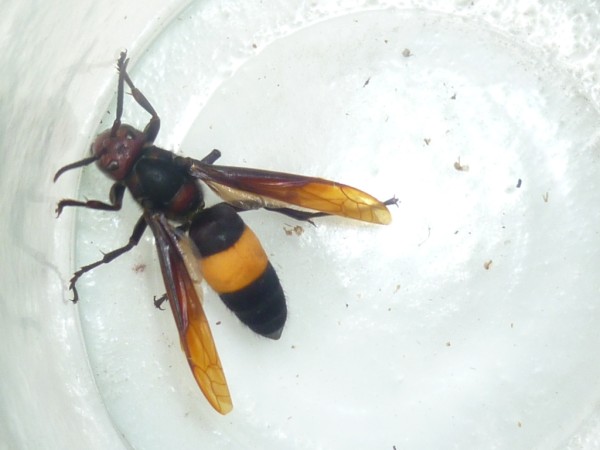
**Hornet, *Vespa affinis.***

The order Hymenoptera represents ants, wasps and bees. The family Vespidae includes stinging social wasps represented by 860 species worldwide. The subfamily Vespinae includes the hornets and yellow jackets [1,2]. The common and widely distributed hornet in Sri Lanka is *Vespa affinis* or *Debara* in Sinhala which belongs to the genus *Vespa*[3]. Hornets construct their nests on tall structures, near or on buildings, under eaves of roofs, or in natural nesting sites such as trees, shrubs and rock faces (Figure 1). The length of a hornet ranges from 2 to 3cm and its mid-body has a yellow band separating its brownish red front from its black hind-part (Figure 2). The stinging apparatus in the Hymenoptera represents the modified ovipositor (egg-laying apparatus) and hence the sting ability is present only in females [1,2]. The stinging apparatus is connected to a venom gland and the sting is used as an offensive or a defensive weapon [1]. A hornet can inflict multiple stings because the stinger has no barbs unlike bees and does not get detached when stinging. The venom of a hornet contains a mixture of histamine-releasing factors, enzymes, haemolysins, neurotoxins, vasodilators, vasospastic amines and phospholipase A [4].

Hornet stinging is a common environmental hazard in Sri Lanka. When disturbed, these insects in swarm, attack people in the vicinity causing multiple sting injuries. The majority of victims recover in hospitals, but an unaccountable number of them die due to acute complications which are unpredictable [4]. The histamine-releasing action of the venom during the first contact with a victim is the most common cause of pathophysiology following hornet stings and other reported manifestations were myocardial infarction, multiple-organ failure, myasthenia gravis, mastocytosis and reversible optic neuropathy [3,5-7]. A victim can die within hours to days despite treatment and very often attempts are not made to understand the pathogenesis of the complications of these unfortunate cases. Thus, performing necropsies and histopathological examinations in sting-related deaths are of paramount importance to understand the pathological processes of fatal cases. We report a case study of two Sinhalese patients, a husband and wife, who died after a massive attack by hornets despite early admission to hospitals within half an hour of the accident. The relatives of the patients later produced dead hornets collected from the site of the accident and they were identified as *Vespa affinis* or *Debara.*

## Case presentations

### Case 1

A 46-year-old Sinhalese woman went to the nearby wood to collect firewood at approximately 1 p.m. in the afternoon and pulled a branch of a tree without noticing a hornet nest attached to it. She then came under massive attack by hornets until her husband came to rescue her. Within half an hour of the attack she was admitted to a local hospital where she was found to have a blood pressure of 90/50mmHg. She received initial resuscitation with isotonic saline and medications such as hydrocortisone, chlorpheniramine maleate and promethazine and she was transferred to the nearest tertiary care hospital in 3 hours. At that time her blood pressure was 138/83mmHg and her radial pulse rate was 114 beats per minute. She was not in respiratory distress and her lungs were clear. An indwelling catheter was inserted that drained 250mL of normal colour urine. At 6 p.m., 5 hours after she was stung, her blood pressure was 160/115mmHg, her pulse rate was 100 beats per minute, her oxygen saturation was 98% and she was drowsy with a Glasgow coma scale of 11/15. She was given another dose of hydrocortisone and chlorpheniramine maleate. Her serum sodium was 136mmol/L, potassium was 3.0 mmol/L, and an electrocardiogram (ECG) showed sinus tachycardia. At 7.30 p.m., 6.5 hours after she was stung, she became oliguric and passed blood-stained urine and became restless. At 9 p.m., 8 hours after she was stung, she became dyspnoeic with a respiratory rate of 32 breaths per minute. Her radial pulse rate was 96 beats per minute, her blood pressure was 86/50mmHg and she had central cyanosis and fine crepitations in her lungs suggestive of pulmonary oedema. She was given high-flow oxygen via a face mask, an intravenous dose of frusemide and infusion of dobutamine while awaiting an intensive care bed. However, in the next 15 minutes she developed a cardiac arrest and underwent continuous resuscitation including intubation and assisted ventilation until she was pronounced dead at 10 p.m. Prior to this incidence she was in good health and was not taking any medication including beta-blockers or angiotensin-converting enzyme (ACE)-inhibitors. At autopsy, her skin had 40 sting marks distributed in her face, neck, chest, abdomen and limbs which were circumscribed and punctated. More than 100mL of blood-stained fluid was found in each pleural space and her lungs were oedematous and showed frothy fluid. Her kidneys were congested. Her myocardium was pale and her coronary arteries were normal. No abnormalities were found in other organs including her brain.

### Case 2

**Figure 3:**
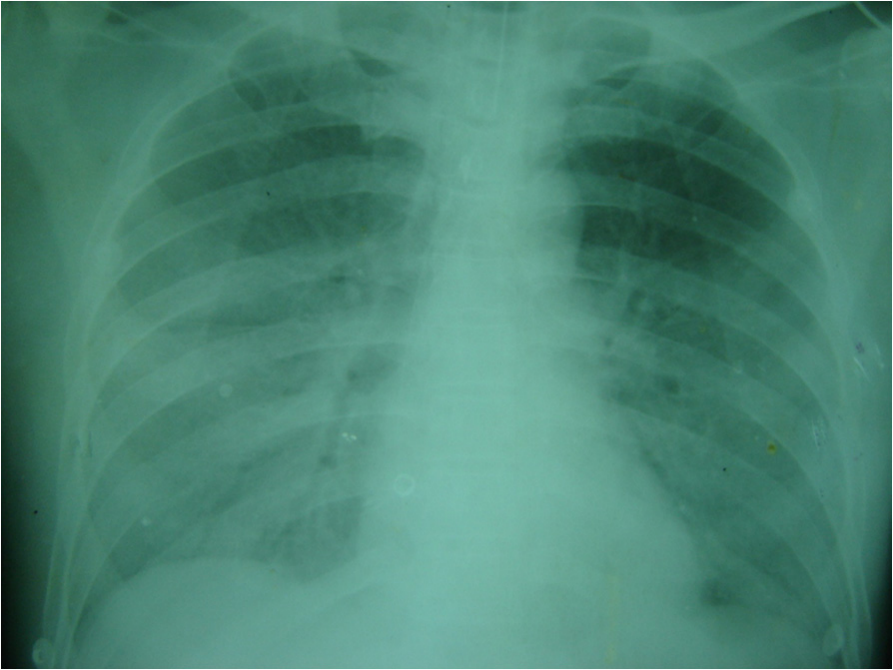
Chest X-ray of Case 2 showing pulmonary oedema.

**Figure 4:**
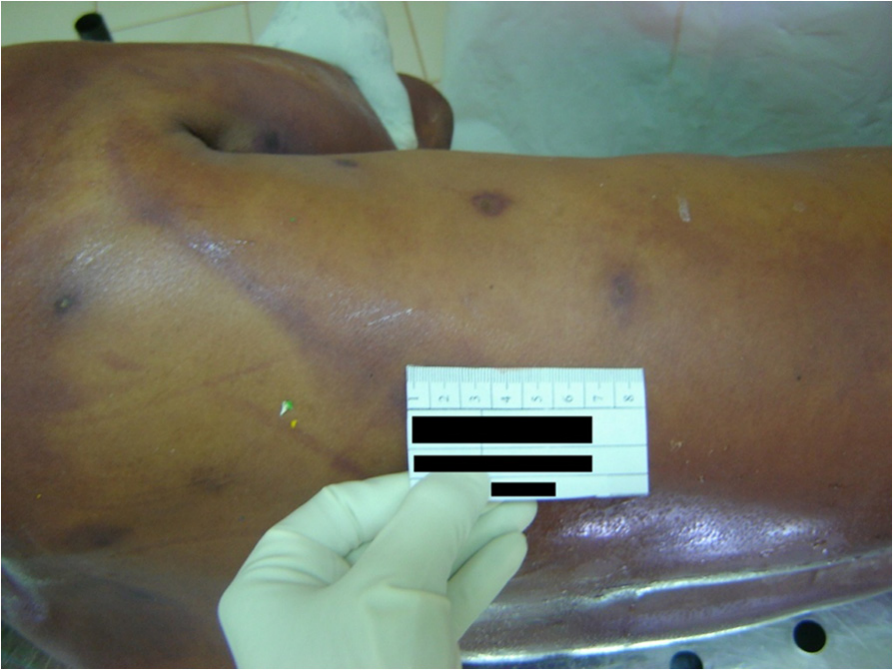
Multiple sting sites with circumscribed punctated sting marks.

**Figure 5:**
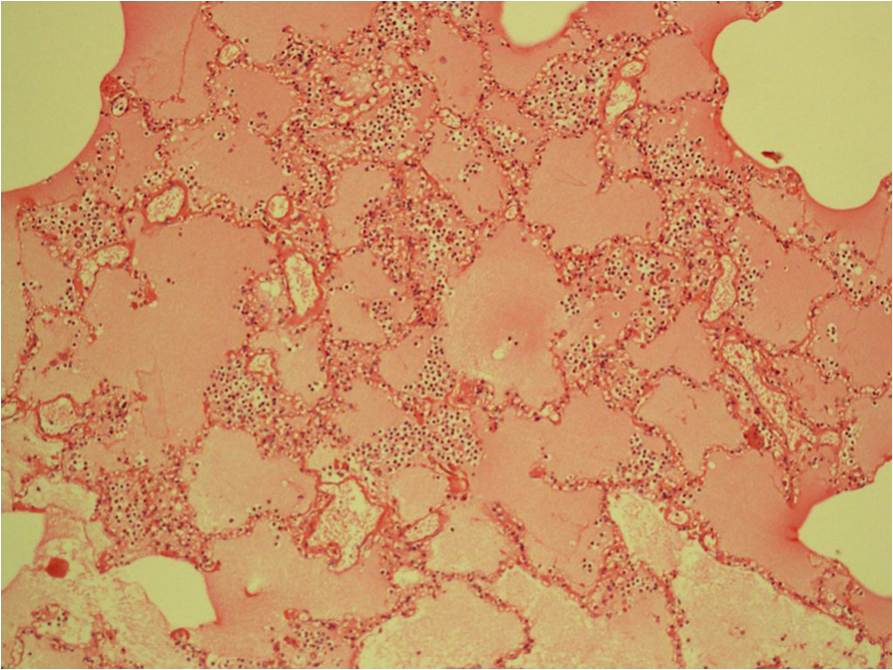
**The alveoli of the lungs filled with thin proteinaceous fluid along with extravasated eosinophils and neutrophils (staining; haematoxylin and eosin)** ***.***

**Figure 6:**
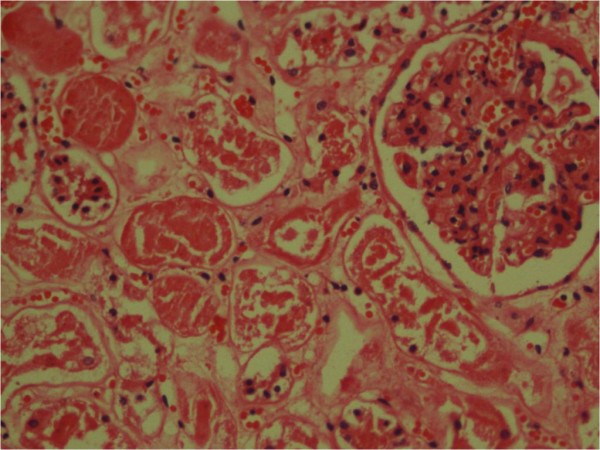
**Renal tubules showing acute tubular injury with red cell casts (staining; haematoxylin and eosin)** ***.***

**Figure 7:**
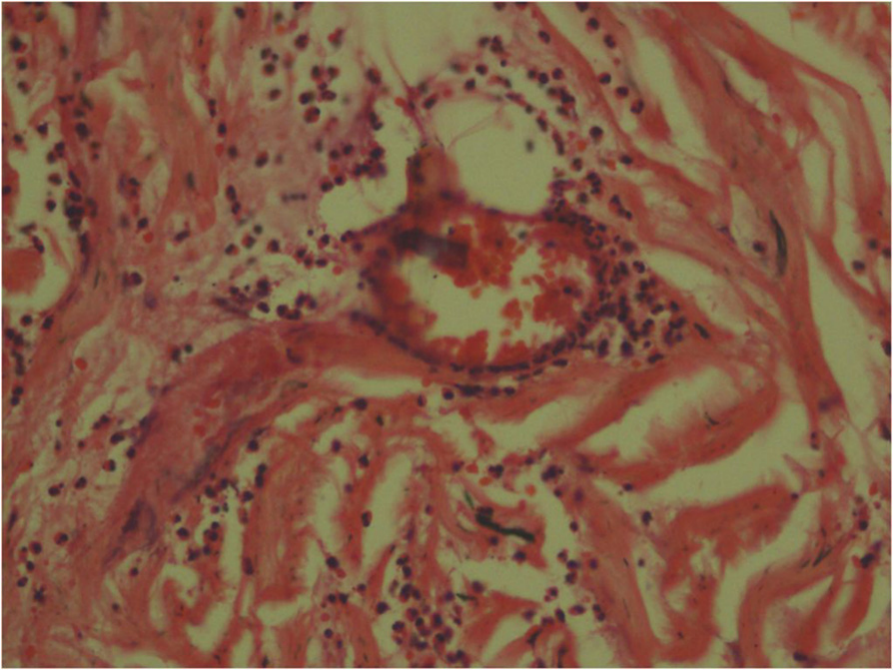
**Postcapillary venules of the skin showing extravasated eosinophils and mast cells (staining; haematoxylin and eosin)** ***.***

**Figure 8:**
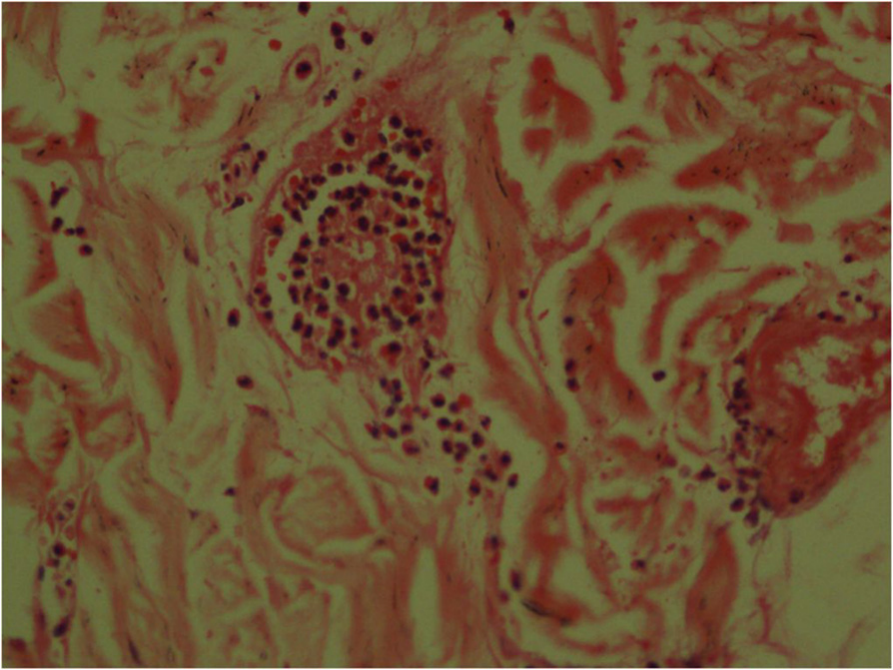
**Skin capillaries congested with eosinophils, mast cells and neutrophils (staining; haematoxylin and eosin)** ***.***

The husband of Case 1 who is a 48-year-old Sinhalese man became a victim of a massive attack by hornets when he tried to rescue his wife. He was admitted to the same local hospital within half an hour of the attack and received the same medications as his wife had received. However, his blood pressure was 80/50mmHg and his pulse rate was 88 beats/minute and his lungs were clear. He was also transferred to the same tertiary care hospital along with his wife and on admission his blood pressure was 130/73mmHg, his pulse rate was 84 beats per minute, his oxygen saturation was 96% and his ECG was normal. He was in pain, but had stable clinical parameters. He started to pass blood-stained urine at 5 p.m., 4 hours after he had been stung, and he was mildly breathless with a respiratory rate of 22 breaths per minute. At 10 p.m., 9 hours after he had been stung, his blood pressure was 80/40mmHg, pulse rate was 90 beats per minute, respiratory rate was 38 breaths per minute and oxygen saturation dropped to72%. He was managed in the intensive care unit where he received inotropic drugs, high-flow oxygen and steroids. At 3 a.m., 14 hours after he had been stung, he became totally anuric and hypoxic, which required continuous mandatory assisted ventilation with 5mmHg positive end-expiratory pressure and he subsequently started peritoneal dialysis. His chest X-ray showed evidence of pulmonary oedema (Figure 3). Despite multipronged supportive care such as assisted ventilation, inotropic drugs, intravenous hydrocortisone and oxygen his blood pressure and oxygen saturation did not improve and he died 32 hours after he had been stung. His initial haemoglobin and pack cell volume were 15g/dL and 43% respectively which rose to 19g/dL and 56% 14 hours after he had been stung. At that time his platelet count was 30 ×10^9^/L, blood urea was 48mg/dL, clotting time was over 10 minutes, international normalised ratio (INR) was 2, pH was 6.95, serum potassium was 5.3mmol/L, serum sodium was 143mmol/L and he was positive for D-dimer. Prior to this incidence he was in good health and was not taking any medication including beta-blockers or ACE-inhibitors. The autopsy examination found approximately 130 circumscribed punctated sting marks distributed all over his body (Figure 4). His chest cavity had more than 150mL of blood-stained fluid in each side and he had 10mL of blood-stained pericardial effusion. His lungs were congested and he had a pale myocardium with normal coronary arteries. His kidneys had pale cortical surfaces and congested medulla.Both patients had similar histological findings of tissues as follows. In the lungs, the alveolar tissues were filled with thin proteinaceous fluid with extravasated eosinophils and neutrophils leading to extensive pulmonary oedema (Figure 5). The kidneys showed a full red cell cast in renal tubules and acute tubular injury (Figure 6). The skin biopsy showed features of dermal oedema, congested postvenular capillaries and extravasated mast cells, eosinophils and neutrophils accounting to hypersensitivity vasculitis (Figures 7 and 8). The histology of their brains, hearts, spleens and adrenal glands was unremarkable.

## Discussion

An unfortunate story of this nature highlights a social issue in which children loose both parents unexpectedly in an environmental accident. Also it proves the aggressive behaviour of disturbed hornets from which the victims find no way of escape. Hornets are ubiquitous in Sri Lanka, nesting close to human dwellings resulting in a constant conflict with humans. This results in immense human suffering and even leads to fatalities. Therefore, priority should be given to recognize hornet stings as an important health issue and to improve management strategies in hospitals thereby reducing the number of deaths. This needs the building up of a thorough knowledge base about possible complications of hornet stings, understanding of venom effects on body homeostasis and testing of different therapeutic regimens that would reverse the deranged physiological processes in the body. In this case study, both patients had similar macroscopic and histopathological findings suggesting an initiation of the same pathophysiological process as a result of envenoming that has progressed to an irreversible outcome.

In both patients, acute pulmonary oedema had been the dominant manifestation detected both clinically and histopathologically that had contributed to their death within a short hospital stay. It is apparent that the development of pulmonary oedema took a few hours in both patients. However, fluid shift as a result of immediate anaphylaxis or anaphylactoid reaction would have started very early leading to the development of acute pulmonary oedema followed by a chain of events such as prolonged hypoxaemia, metabolic acidosis and cardiac dysfunction. During the early hours, onset of acute pulmonary oedema was not obvious clinically and detected later in advanced stages when the damage was irreparable. In the literature, wasp venom-induced massive acute pulmonary oedema has been reported in a 38-year-old Chinese woman in whom the causative mechanism was unexplained [8]. In such situations with normal cardiac status, the only explanation that can be given is increased microvascular permeability in the lungs causing extravasations of fluid into alveolar spaces causing acute pulmonary oedema. A possibility of massive fluid shift was very high in both patients as they also had pleural effusions and in Case 2, there was increased haemoglobin and pack cell volume suggesting haemoconcentration as a result of plasma leak. We need to understand the causative mechanism of fluid leaking out of microcirculation and both toxicokinetics and toxicodynamics.

In a series of 56 deaths after anaphylaxis studies in the UK, 23 cases had nothing indicative of an allergic pathology, and were attributed to severe shock secondary to anaphylaxis [9]. The same study compared clinical and postmortem findings between three causes of anaphylaxis namely venom, food and drugs. Drugs have caused anaphylaxis in a mean time of 5 minutes whereas venom and food taken more than 20 minutes. All aetiological causes have caused immediate deaths, mucous plugging of bronchi, and pulmonary congestion in most of the cases. However, distinct differences were that food allergic reactions caused more breathing difficulties whereas venom caused shock without breathing difficulties in many cases [9]. These findings highlight that there could be differences of dominance of pathological processes between causes of anaphylaxis, and pulmonary manifestation could occur in venom-induced anaphylaxis.

Other than acute pulmonary oedema, development of acute venom-induced consumptive coagulopathy or disseminated intravascular coagulation occurred in Case 2 as suggested by increased blood clotting time, INR, thrombocytopenia and raised D-dimers in his blood. Both patients had developed acute kidney injury that was preceded by passing red-coloured urine. However, initial output and the colour of the urine were normal in the first patient. Microscopic finding of renal tissues showed red cell casts and tubular injury. Acute kidney injury and accumulation of toxic metabolites would have contributed to pulmonary oedema, myocardial dysfunction and metabolic acidosis and all these factors would have contributed to the fatal outcome. In Case 1, she had a cardiac arrest within a short time after development of pulmonary oedema. But in Case 2, the patient deteriorated over hours with multiple organ dysfunction and metabolic derangements. Multiple organ dysfunction and acute kidney injury has been reported after wasp stings [8,10-12]. Usually these complications are irreversible, except in a rare case of recovery after being anuric for 5 days [3].

The immune mechanisms causing the histamine-releasing action of Hymenoptera stings are either Type 1 hypersensitivity that operates through immunoglobulin E-mediated mast cell degranulation leading to anaphylaxis or anaphylactoid reaction where immunoglobulins are not involved [3,5] or, occasionally, delayed reactions due to Type III hypersensitivity immune response that could cause Arthus reaction and serum sickness. It is apparent that deaths happening a few hours or days later could be due to different problems which are unpredictable. Other than lungs and kidneys, other organs did not show significant histopathological changes in these patients. On microscopic examination the myocardium and coronary arteries were normal and what caused the cardiac arrest was not ascertained. Finally, acute pulmonary oedema and increased microvascular permeability should be the main pathology that contributed to these deaths. Whether this could have been averted by use of adrenaline and meticulous fluid management at the outset needs to be addressed. Lessons learnt reiterate that immediate recognition of anaphylaxis, early use of adrenaline, inhaled beta agonist and other measures are crucial for a successful outcome [13]. Therefore, the positive impact of adrenaline administration in the early stage of Hymenoptera envenoming is clear irrespective of either anaphylactic or anaphylactoid reaction.

By doing critical analysis of these fatal cases one might find possible lapses in the management of them such as insufficient monitoring, missing onset of organ dysfunctions, delays in early intensive care and organ support and inappropriate therapeutic decisions and medications. Lack of standard management guidelines of Hymenoptera envenoming is a global problem which has an amplified effect on victims in resource-poor countries. Therefore, vigilance is needed from the time of stinging to detect all the complications and to institute proper management to increase the chances of survival of the victim. The severity of envenoming and the late complications are related to the number of stings as shown in these cases, but sometimes a single sting could be fatal in a sensitised individual. Hymenoptera stings and envenoming needs more global attention as it appears to be a neglected problem now.

## Conclusions

Early recognition of acute pulmonary oedema in hornet stings is needed with implementation of crucial treatments such as early use of assisted ventilation, organ support, dialysis, and medications such as adrenaline to avert deaths. Further studies are needed to understand the pathophysiological mechanisms of pulmonary oedema in Hymenoptera envenoming and to find out appropriate preventive treatments.

## Consent

Written informed consent was obtained from the patients’ next-of-kin for publication of this case report and accompanying images. A copy of the written consent is available for review by the Editor-in-Chief of this journal.

## Abbreviations

ACE: Angiotensin-converting enzyme; ECG: Electrocardiogram; INR: International normalised ratio.

## Competing interests

The authors declare that they have no competing interests.

## Authors’ contributions

SAMK, KK and TK conceived and designed the study. AJK and KW collected data. AJN did autopsies and RW did histopathological studies. SAMK and RW drafted the report. All authors contributed to intellectual content, review and revision of the report, and have seen and approved the final version.
